# Integrated Multi-Omics Perspective to Strengthen the Understanding of Salt Tolerance in Rice

**DOI:** 10.3390/ijms23095236

**Published:** 2022-05-07

**Authors:** Liping Dai, Peiyuan Li, Qing Li, Yujia Leng, Dali Zeng, Qian Qian

**Affiliations:** 1 State Key Laboratory for Rice Biology, China National Rice Research Institute, Hangzhou 310006, China; dailiping777@126.com (L.D.); 15109318310@163.com (P.L.); liqing1986102@163.com (Q.L.); dalizeng@126.com (D.Z.); 2 Jiangsu Key Laboratory of Crop Genomics and Molecular Breeding/Key Laboratory of Plant Functional Genomics of the Ministry of Education/Jiangsu Key Laboratory of Crop Genetics and Physiology/Jiangsu Co-Innovation Center for Modern Production Technology of Grain Crops, College of Agriculture, Yangzhou University, Yangzhou 225009, China; 3 The Key Laboratory for Quality Improvement of Agricultural Products of Zhejiang Province, Zhejiang A & F University, Hangzhou 311300, China

**Keywords:** rice, salt stress, omics, salt tolerance

## Abstract

Salt stress is one of the major constraints to rice cultivation worldwide. Thus, the development of salt-tolerant rice cultivars becomes a hotspot of current rice breeding. Achieving this goal depends in part on understanding how rice responds to salt stress and uncovering the molecular mechanism underlying this trait. Over the past decade, great efforts have been made to understand the mechanism of salt tolerance in rice through genomics, transcriptomics, proteomics, metabolomics, and epigenetics. However, there are few reviews on this aspect. Therefore, we review the research progress of omics related to salt tolerance in rice and discuss how these advances will promote the innovations of salt-tolerant rice breeding. In the future, we expect that the integration of multi-omics salt tolerance data can accelerate the solution of the response mechanism of rice to salt stress, and lay a molecular foundation for precise breeding of salt tolerance.

## 1. Introduction

High salinity in the soil is one of the most harmful environmental stresses to the growth and development of plants. Its toxicity to plants is mainly displayed in four aspects: (1) osmotic stress: when the soil salinity increases, the water potential of soil solution will be lower than that of plant root cells, which makes root absorption difficult and causes osmotic stress; (2) nutritional stress: osmotic stress can cause stomatal closure of plants, inhibit the absorption of CO_2_, thereby weakening photosynthesis and causing nutritional deficiency; (3) ion stress: the accumulation of Na^+^ and Cl^−^ in cells affects the absorption and transport of mineral elements such as K^+^, Ca^2+^ and inhibits the activity of enzymes in cells; (4) Reactive oxygen species (ROS) accumulation: salt stress can lead to the accumulation of reactive oxygen species, thereby breaking cell structure and biological macromolecules [1,2,3]. It is estimated that over 1/3 of irrigated lands in the world are affected by salinization [4], and this ratio is expected to rise due to the increasing climate change, seawater intrusion, and poor-quality agricultural management practices [5,6]. Therefore, soil salinization becomes a worldwide problem that severely reduces global crop yield and agricultural production [7,8].

Rice (*Oryza sativa* L.), as the staple food for more than half of the world’s population [9,10], is a glycophyte and more susceptible to high salinity [2,3]. Increasing soil salinization has seriously restricted rice planting and production, thus threatening food security [11]. Therefore, breeding salt-tolerant rice and farming in saline-alkali land is an effective strategy to ensure food security in the face of the increasing human population. To achieve this goal, it is necessary to understand how the morphological, physiological, and biochemical properties of rice respond to high salinity and elucidate the regulatory mechanisms underlying these traits. Given the importance of the salt tolerance mechanism in rice, it has been extensively studied. To date, more than 85 QTLs and genes related to rice salt tolerance have been identified by conventional QTL mapping and map-based cloning from mutants, which have been summarized by several recent reviews [10,12,13]. Among these QTLs or genes, *SKC1* is the first isolated salt stress-related QTL in rice, which encodes a sodium ion (Na^+^) transporter of the HKT family [14]. *OsNHX1*, encoding a vacuolar Na^+^/H^+^ antiporter, plays a key role in the transport of Na^+^ highly accumulated in the cytoplasm into the vacuole [15]. The plasma membrane Na^+^/H^+^ exchanger protein OsSOS1 is the only determining Na^+^ efflux transporter, which can be activated by the OsCBL4-OsCIPK24 complex [16,17]. *OsAKT1* and *OsAKT2* encoded potassium channel proteins that are essential for the absorption of potassium ions (K^+^) by rice roots. The OsAKT1 or OsAKT2-mediated K^+^ uptake was further enhanced by the OsCBL1-OsCIPK23 complex [18,19,20].

Despite a series of QTLs and genes associated with salt tolerance in rice having been characterized, our understanding of the regulatory network of salt response remains limited. Fortunately, with the rapid development of high-throughput technology and bioinformatics, the research on rice responses to salt stress has undergone unprecedented changes and remarkable advances over the past decade. In this paper, we reviewed the studies on genomics, transcriptomics, proteomics, metabolomics, and epigenetics related to salt stress to deepen the understanding of salt tolerance in rice. In this process, we also recognize that omics-based approaches also have limitations. Given the complexity of the salt stress response process, we believe that current and future efforts should focus on the integration of multi-omics data and the introduction of new technologies (such as microbiology, phenomics, and artificial intelligence) to overcome the existing problems and further explore the molecular mechanism of salt stress response in rice, and lay a theoretical foundation for cultivating salt-tolerant rice varieties.

## 2. Rapid Isolation of Candidate Loci or Genes for Salt Tolerance Using Whole-Genome Sequencing

### 2.1. Identification of Salt-Tolerant Loci by BSA-Seq

BSA-seq is a bulked segregant analysis (BSA) technology based on whole-genome resequencing approaches [21]. Compared to conventional methods such as QTL- or map-based cloning using individual markers, BSA-seq is a fast and cost-effective approach for mapping qualitative or quantitative trait loci [22]. The bulks for BSA-seq can be established by the selection of extremes or representative samples from any population and all types of segregants that reflect broad phenotypic variation for the target trait [23]. According to the differences in bulked sample sources and sequencing strategies, BSA-seq can be divided into MutMap series (MutMap, MutMap^+^, MutMap-gap), and QTL-seq. MutMap series is mainly applicable to populations from mutant materials [24], while QTL-seq is suitable for populations derived from natural materials [25].

As a rapid QTL and gene identification method, BSA-seq has been successfully used to detect potential salt tolerance loci in rice. Takagi et al., first used the MutMap method to identify the gene conferring salt tolerance in rice and found *OsRR22* is responsible for the salinity-tolerance phenotype of *hst1* rice [26]. However, due to the lack of salt stress-related mutants, the application of MutMap for salt tolerance in rice is limited. Compared with MutMap, QTL-seq with natural material sourced population is more widely used. Tiwari et al. identified 21 salt-tolerant QTL in the reproductive stage of rice using the QTL-seq method based on bi-parental recombinant inbred lines (RIL) with extreme salt tolerance phenotype [27]. Using the salt-tolerant and salt-sensitive salinity bulks of F_2_ population derived from the cross between Zhefu 802 (a cultivar sensitive to salt) and Changmaogu (a novel, strongly salt-tolerant rice landrace), Sun et al. mapped six candidate regions for salt tolerance at the seedling stage in rice by QTL-seq [28]. Depending on the polymorphisms identified between Zhefu 802 and Changmaogu, they further found 32 genes containing nonsynonymous SNPs and frameshift mutations in the open reading frame (ORF) regions. In addition, two major salt-tolerant QTLs, *qST1.1* and *qRSL7*, that might contribute to the salt tolerance of “Sea Rice 86” and “Weiguo” respectively, were determined similarly [29,30].

### 2.2. Identification of Salt-Tolerant Loci by GWAS

Genome-wide association study (GWAS) uses statistical methods to look for the associations between sequence polymorphisms and phenotypic variation among accessions. It has proved to be a powerful way to dissect complex traits in plants. Compared with traditional QTL mapping using a bi-parental population, GWAS has two major advantages. First, the GWAS population has more natural variation than the segregation populations derived from the two parental lines. Second, most GWAS can produce a relatively high mapping resolution due to the existence of diverse historical recombination events and high-density SNP markers. In the past decade, GWAS has been successfully used to detect the potential loci for salt tolerance at various developmental stages in rice, and several important genes were identified and further validated by functional experiments.

Seed germination is the first step in determining crop yield. Increasing the salt tolerance of direct-seeding rice during the seed germination stage is a major breeding goal for many breeders. Therefore, it is of great significance to understand the genetic mechanisms of salt tolerance in rice at the seed germination stage. Shi et al., surveyed seven seed germination-related traits of 478 re-sequencing rice accessions under salt stress and performed a GWAS based on 6,361,920 SNPs in these accessions [31]. They identified eleven loci based on the stress-susceptibility indices of vigor index and mean germination time, among which seven were consistent with the previously found resistance-related loci. Cui et al., detected 371 quantitative traits nucleotides (QTNs) for the salt tolerance traits at the seed germination stage by six multi-locus GWAS methods, 56 of which were identified by at least three methods [32]. In addition, 66 candidate genes were found in the vicinity of these 56 QTNs, among which *LOC_Os01g45760* and *LOC_Os10g04860* are involved in auxin biosynthesis. Yu et al., explored salt tolerance in 295 rice accessions at the germination stage, and a total of 12 associated peaks and 79 candidate genes were detected by GWAS [33]. By detecting highly correlated variations in genic regions that overlapped with linkage disequilibrium (LD) block analysis, they further identified 17 genes that might contribute to salt tolerance at the germination stage. Naveed et al., evaluated the salt tolerance-related traits of 208 rice mini-core accessions at both germination and seedling stages, and detected six QTNs for salt tolerance at the germination stage, three of which locate within the vicinities of three cloned salt tolerance-related genes, *OsGMST1*, *DSM3,* and *OsCCC1* [34].

The rice seedling stage is sensitive to salt stress, and thus this stage is the best time to identify salt tolerance in rice. For example, Meyer et al., identified 11 significant loci for six salt tolerance traits at the seedling stage through GWAS mapping with the 93 African rice accessions, four of which were located within ~300 kb of positive selected genomic regions, suggesting adaptive geographical differentiation for salt tolerance in these accessions [35]. Yu et al., conducted a GWAS of salt tolerance-related phenotypes at the seedling stage in 295 rice accessions, and detected 93 candidate genes with high association peaks, among which 33 genes were associated in a protein interaction network [36]. Frouin et al., evaluated the salt tolerance of 231 temperate *japonica* rice accessions at the seedling stage, and a total of 73 QTL were identified by GWAS. The locations of some QTLs were close to those of genes related to calcium signaling and metabolism, suggesting that the calcium signal-mediated ion homeostasis plays a key role in regulating the salt tolerance of temperate rice [37]. Batayeva et al., identified 26 QTLs for nine salt tolerance traits at the seedling stage by GWAS in 203 temperate *japonica* rice accessions, 11 of which were co-located with known salinity tolerance genes [38]. Based on GWAS in 306 rice accessions, Patishtan et al., found the salt tolerance of these seedlings was associated with the ubiquitination pathway [39]. An et al., detected 54 QTLs associated with salt tolerance at the seedling stage through GWAS using 181 rice accessions. In a novel QTL *qst-7.19*, the strong candidate gene, *LOC_Os07g31884*, encoding a multidrug and toxic compound extrusion (MATE) protein was further validated for salt stress using single chromosomal segment substitution lines (CSSLs) [40]. The GWAS of Rohila et al., identified 9 QTLs associated with salt tolerance at the seedling stage using USDA rice mini-core collection (URMC), of which none were in the known salt-tolerant QTL region, implicating different genes and mechanisms responsible for salt tolerance in the URMC [41]. Yuan et al., performed GWAS with different analytical models in 664 rice cultivars, and identified 21 QTLs and two functional candidate genes, *OsSTL1* and *OsSTL2*. Sequence analysis showed that *OsSTL1* was homologous to salt tolerance gene *SRP1* in *Arabidopsis* [42]. Chen et al., carried out GWAS for salt tolerance of rice seedlings grown in both hydroponic and soil systems using 204 *aus* accessions, and detected 97 and 74 QTLs associated with traits in hydroponic and soil respectively, including 11 QTLs identified in both systems. The well-known Na^+^ transporter gene *OsHKT1;5*, and two genes *OsGTγ-1* and *OsCPK13* involved in salt-stress were co-localized with these QTLs in rice [43]. Nayyeripasand et al., identified 29 genome regions associated with 12 salinity-related traits at the early vegetative stage of 155 rice accessions, a genomic region of which was co-located with a major QTL *SalTol1* for salt tolerance at the vegetative stage [44]. The GWAS of Yadav et al., detected 23 marker-trait associations (MTAs) in 96 rice accessions, 21 of which were co-located at or close to the reported QTL loci, and the remaining two, named *qSDW2.1* and *qSNC5*, were newly discovered [45]. Alternative splicing (AS) refers to a gene that produces multiple different mature mRNAs by RNA splicing during the process from mRNA precursor to mature mRNA, and is an essential transcriptional and post-transcriptional regulatory mechanism in plant response to abiotic stresses. Yu et al., developed a software tool to visualize and quantify the variations of splicing in population (VASP), and identified 764 significant genotype-specific splicing (GSS) events in an RNA-seq database from a rice diversity panel exposed to normal and saline growing conditions. Using GSS events as markers of GWAS, they found six GSS events in five genes significantly associated with the shoot Na^+^ content in rice seedlings. Among them, *OsRAD23* and *OsNUC1* were the preferred candidate genes with splice variants that showed a significant difference between the variants for shoot growth under salt stress [46].

Similar to the seedling stage, the rice reproductive stage is also extremely sensitive to salinity, and salt tolerance in this period is an important factor in determining rice yield. Thus, to maximize rice yield in salinized soil, it is urgent to discover new salt-tolerant genes at the reproductive stage and introduce them into salt-sensitive rice cultivars. Kumar et al., conducted GWAS to identify loci controlling salinity tolerance at the reproductive stage in 220 rice accessions, and detected 64 significant SNPs under salt stress. The region containing *Saltol*, a major QTL for salt tolerance at the vegetative stage, was identified as a major association with Na^+^/K^+^ ratio determined at the reproductive stage, suggesting that *Saltol* is an important QTL for rice salt tolerance [47]. Lekklar et al., implemented GWAS for salinity tolerance at the flowering stage based on exome sequencing of 104 Thai rice accessions, and detected 448 significant SNPs in the exome of 200 genes. Among these, there were 146 genes, which accounted for 73% of the identified candidate genes, co-localized with the previously reported salt QTLs. While many of these are novel, their annotations are consistent with potential involvement in salt tolerance and related agronomic traits [48]. In a GWAS using 708 rice accessions, Liu et al., identified 2255 marker-trait association signals for salt tolerance and yield-related traits, and the significant QTNs were distributed in 903 genes. Further haplotype analysis revealed 15 strong candidates significantly associated with the target traits, including 10 novel genes and five known genes (*OsGAMYB*, *OsMYB6*, *OsHKT1;4*, *OsCTR3*, and *OsSUT1*) [49]. In addition, by GWAS for salinity tolerance at reproductive stage in 180 rice accessions, Warraich et al., successfully identified 28 significant marker-trait associations, out of which 19 were identified for Na^+^, K^+^, Na^+^/K^+^ uptake in rice. The position of some associated markers were close to candidate genes, such as transcription factors, signal transducers, membrane transporters, which have previously been shown to play positive roles in rice salinity tolerance [50].

**Figure 1:**
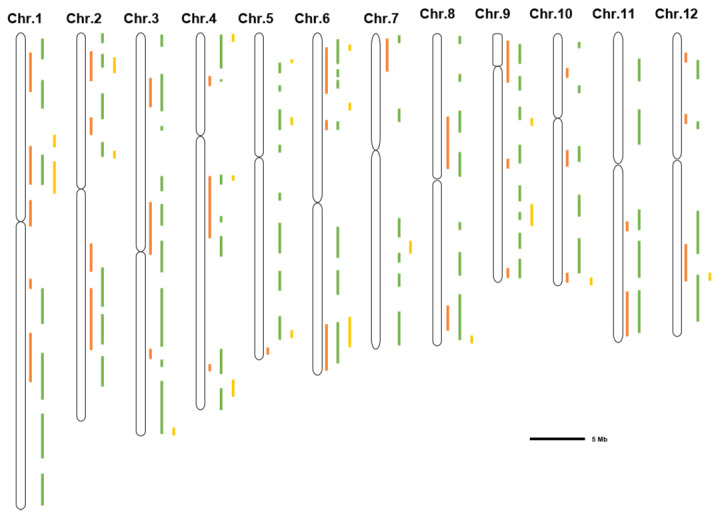
Distribution of salt stress-related QTLs/QTNs detected by GWAS on 12 chromosomes (Chr) of rice. The 12 long columns represent the 12 chromosomes of rice, respectively. The orange, green and yellow bars represent QTLs/QTNs positions detected at the stages of germination [31,32,33,34], seedling [34,35,36,37,38,40,41,42,43,45,46,51,52] and reproductive [47,48,49,50], respectively. The length of the black line in the lower right corner of the figure represents the physical distance of the chromosomes.

A summary of the salt tolerance-related loci showed that some loci were detected in different stages, such as the loci of 11.5 Mb on chr.1 [31,32,34,37,43,45,47,50], 4.2 Mb on chr.3 [32,38,42,51], 7.5 Mb on chr.5 [40,50,52], 20.8 Mb on chr.7 [40,50], and 21.0 Mb on chr.9 [32,37,40]. In addition, each period has its unique loci. For example, the loci of 42.0 Mb on Chr.1 [37,43], 34.0 Mb on Chr.2 [37,42,43,51], 22.0 Mb on Chr.6 [36,37,38,40,43], 0.5 Mb on Chr.8 [37,40,43,51], 15.0 Mb on Chr.10 [34,36,37,38], and 26.0 Mb on Chr.12 [36,51] (Table 1; Figure 1). These loci and candidate genes provide valuable resources for validating potential salt-tolerant genes and will promote rice breeding for salt tolerance via marker-assisted selection.

## 3. Genome-Wide Expression Analysis Is Helpful to Elucidate the Molecular Mechanism of Salt Tolerance

Genome-wide gene expression study based on transcriptomic techniques is an effective approach for dissecting the gene regulatory networks of complex agronomic traits. Transcriptomics can be divided into cDNA microarray-based on hybridization and RNA-seq that rely on next-generation sequencing. cDNA microarray is designed to fix a gene probe on a solid phase device through micromachining technology, and then hybridize with the labeled sample for analysis by detecting the hybridization signal. cDNA microarray is a process in which predesigned gene probes are fixed to solid-phase devices using micromachining techniques and then hybridized with labeled samples to quantify gene expression by detecting hybridization signals. But cDNA microarray could not detect new transcripts due to the predesigned templates, and the results were inaccurate when the gene expression was too low or too high. In contrast, RNA-seq is a high-throughput sequencing technology that can not only reflect the expression levels of mRNA, small RNA, noncoding RNA, but also discover new transcripts or even a new gene, and thus it is more commonly used than cDNA microarray at present.

### 3.1. mRNA Response to Salt Stress

The molecular mechanism of plant response to salt stress involves multiple processes, including sensing, signal transduction, transcription, transcription processing, translation, and post-translational modifications [53]. Thanks largely to the high-throughput transcriptome detection technology for rice, cDNA microarray or RNA-seq has been successfully applied to monitor global gene expression changes responsive to salt stress in rice. Furthermore, several key genes were identified and further validated by functional experiments.

Plants growing in saline soils are subjected to Na^+^-induced ionic stress. To avoid Na^+^-induced ionic stress, higher plants develop a series of ion transport mechanisms to maintain Na^+^ homeostasis. Through a microarray analysis in eight rice genotypes with a wide natural variation in terms of taxonomy, origin and salt sensitivity, Hossain et al., identified about 60 genes that were involved in Na^+^, K^+^, and anion homeostasis, transport, and transmembrane activity under salt stress [54]. Mansuri et al., compared transcriptome profiles of the salt-tolerant recombinant inbred line FL478 and its salt-sensitive parent IR29 by RNA-seq. The results showed that FL478 responded to salt stress through a series of sophisticated mechanisms, one of which was proficient influx and transport of K^+^ via up-regulating the expression of several potassium transporter encoding genes such as *HAK6*, *HAK25,* and *RCN1* [55]. Fu et al., reported that *OsC2DP*, encoding a novel C2 domain-containing protein, was important for salt tolerance in rice [56]. *OsC2DP* was preferentially expressed in the roots and its expression was significantly down-regulated under salt stress according to rice root transcriptomic analysis [57]. Knockout of *OsC2DP* improved shoot Na^+^ concentration and resulted in rice hypersensitivity to salt stress. What’s more, the quantitative Real-time PCR and RNA-seq analysis showed that the expression levels of *OsSOS1* and *OsNHX4* related to Na^+^ or K^+^ transport were indirectly regulated by *OsC2DP* under salt stress [56]. Wei et al., found that *OsPRR73* can positively regulate salt tolerance in rice. RNA sequencing and biochemical assays revealed *OsHKT2;1*, encoding a plasma membrane-localized Na^+^ transporter, was a transcriptional target of OsPRR73-mediated salt tolerance [58].

Salt stress perception triggers stress-specific signal transduction involving the second messenger ROS. ROS, including superoxide anion, H_2_O_2_, hydroxyl radical, and singlet oxygen, are harmful to biomolecules when their contents exceed the cellular capacity for detoxification, but they also play a key role in stress signaling at low concentrations. Using microarray analysis, Jan et al., found that OsTZF1, a CCCH-Tandem Zinc Finger Protein, confers salt stress tolerance in rice by regulating ROS homeostasis-related genes and other stress-related genes [59]. By comparing the transcriptome of the two rice genotypes with contrasting salt tolerance, Li et al., found that the expression levels of some ROS scavenging genes related to antioxidant systems, such as genes encoding glutathione S-transferase, metallothioneins, and oxidoreductase, were up-regulated in salt-tolerant rice. While the antioxidant capacity of salt-tolerant rice increased, such responses were lacking or minimal in salt-sensitive rice due to the down-regulation of peroxidases and copper/zinc superoxide dismutase [60]. Melatonin, an antioxidant in both animals and plants, has been reported to have beneficial effects in defense against salt stress in rice [61]. Genome-wide expression profiling by RNA-seq showed that melatonin was an effective free radical scavenger, and its exogenous application can enhance antioxidant protection in rice.

Phytohormones, especially ABA, are of vital importance in salt tolerance [4]. Rabbani et al., identified 57 high salinity-induced and 43 ABA-induced genes by microarray analysis, and 39 genes were induced by both stresses, suggesting a strong cross-talk between signaling pathways of salt stress and ABA stress [62]. Similarly, by comparative transcriptome in two rice genotypes with contrasting salt tolerance under salt stress and salt + ABA conditions, Wang et al., found that the majority of differentially expressed genes (DEGs, salt + ABA treatment compared with only salt stress) were common in both genotypes, many of which were involved in ABA-mediated pathways [63]. Based on transcriptome analysis and functional verification, Dong et al., proposed that salt stress induces SERK2 accumulation to amplify early BR signaling in favor of the anti-stress response [64]. From the transcriptomic analysis using two Italian rice varieties with different salt sensitivity, Formentin et al., detected a large number of genes associated with hormone regulation in response to salt stress. These genes are involved in biosynthesis, signaling, response, catabolism, and interplay of phytohormones such as ABA, cytokinin, auxin, gibberellin, ethylene, BR, salicylic acid, and jasmonic acid. They believed that tolerant varieties had faster regulation of hormone metabolism [65].

Salt stress-induced signal transduction can reprogram genome-wide transcripts to produce protective mechanisms such as osmotic adjustment, detoxification, repair of stress-induced damages, and attenuation or amplification of stress signaling. Stress-specific transcription patterns communicate with upstream signaling through transcription factors. By RNA-seq analysis using the salt-tolerant Dongxiang wild rice, Zhou et al., detected a number of differential expression transcription factors such as the families of *NAC*, *MYB*, *bZIP*, and *AP2*/*ERF*, indicating that transcription factors play an important role in responding to salt stress by regulating the expression of downstream genes in plants [66]. The RNA-seq data of salt-tolerant variety (Mulai) and sensitive variety (IR29) showed that more transporters were found to be involved in salt tolerance regulation in Mulai roots such as those that function in ion and sugar transport, while many transcriptional regulation-related genes were detected in IR29 such as transcription factors of *NAC*, *WRKY*, and *MYB* [67]. Comparative transcriptome analysis identified 30 transcription factors (11 *AP2* family members, 14 HB family members, 3 WRKY family members, 1 B3 domain transcription factors and *OsHsfA7*) with increased expression exclusively in exogenous melatonin-treated rice seedlings under salt stress conditions, implicating these transcription factors may be contributing to melatonin-mediated salt tolerance in rice [68].

Photosynthesis, which is crucial for plant growth and development, is also affected by salt stress. Udomchalothorn et al., found that overexpression of the full-length *OsNUC1* (*OsNUC1*-*L*) gene, encoding rice nucleolin, improved salt tolerance in *Arabidopsis* [69]. Transcriptome analysis of the transgenic lines, in comparison with the wild type, showed that *OsNUC1* inhibited the expression of photosynthesis-related genes under normal conditions, while it increased the expression of genes involved in the light-harvesting complex in salt stress conditions. Correspondingly, the net photosynthesis rate of the transgenic *Arabidopsis* and rice increased under salt stress. Similarly, overexpression of the short-length *OsNUC1* (*OsNUC1-S*) enhanced the photosynthetic rate in transgenic rice under salt stress due to the enriched DEGs related to photosynthetic processes that were revealed by a transcriptome comparison [70]. These results suggest that *OsNUC1* plays a role in transcriptome modification, especially in photosynthesis-related genes, and thus stabilizes photosynthesis under salt stress. Khrueasan et al., compared the transcriptome pathways of salt-sensitive variety KDML105 and its salt-tolerant CSSL for their salt tolerance, and found that the differential genes *OsIRO2* and *OsMSR2* located on salt-tolerant quantitative trait loci (QTLs) in chromosome 1 have the potential to participate in salt stress response, especially *OsMSR2*, whose orthologous gene in *Arabidopsis* has a role in photosynthesis adaptation under salt stress [71].

### 3.2. Small RNAs Response to Salt Stress

Small RNAs (20–40 nucleotides in length) not only participate in the regulation of plant growth and development, but also play an important role in plant stress response through gene silencing at the transcriptional and post-transcriptional levels. Based on their biogenesis and precursor structure, they can divide into two prominent types: endogenous short interfering RNAs (siRNAs) and microRNAs (miRNAs). siRNAs are derived from double-stranded RNA precursors, which can be further divided into natural antisense transcripts-derived siRNAs (nat-siRNAs), trans-acting siRNAs (ta-siRNAs), long siRNAs (lsiRNAs), and heterochromatic siRNAs (hc-siRNAs). miRNAs are distinguished from siRNAs since they are transcribed into a long single-stranded pri-miRNA by RNA polymerase II. The pri-miRNA is first cleaved into a stem-loop miRNA precursor (pre-miRNA), and then the pre-miRNA is processed by DCL1 to produce a double-stranded miRNA-miRNA* duplex. The duplex is further methylated at the 3′ end by HEN1. The methylated guide strand (miRNA) is incorporated into AGO1 and functions as the mature miRNA associated with the RNA-induced silencing complex (RISC) to target mRNAs for cleavage in a sequence-specific manner. In contrast, the passenger strand (miRNA*) of the duplex is usually degraded.

Although some abiotic stress-regulated small RNAs have been identified in plants, there are few reports on the response and regulation of small RNAs to salt stress in rice, especially by small RNA sequencing. Sunkar et al., constructed three small RNA libraries from control rice seedlings and seedlings exposed to drought or salt stress, and identified 58,781 and 80,990 unique genome-matching small RNAs from the control and salt stress libraries respectively by high throughput sequencing [72], suggesting high salinity results in changes in the number or expression of small RNAs. Based on Illumina deep sequencing technology, Barrera-Figueroa et al., identified 10 miRNAs regulated by salt stress in rice inflorescences [73]. Tripathi et al., employed the next-generation sequencing technology (NGS) to detect the expression level of miRNAs in glyoxal glycosidase overexpression salt-tolerant rice, and identified many salt-mediated miRNAs that may play an important role in developing salt tolerance [74]. Parmar et al., identified several differentially expressed miRNAs responsive to salt stress in salt-tolerant Pokkali by high-throughput sequencing, which targeted transcription factors such as AP2/EREBP, NAC, ARF, MYB, NF-YA, TCP, HD-Zip III, and SBP reported to be generally involved in salt tolerance or other abiotic stress tolerance [75]. In addition, one of the obtained novel miRNAs, osa-miR12477, targeted L-ascorbate oxidase (LAO), suggesting the accumulation of oxidative stress in rice upon salt stress [75]. Based on a comparison of miRNA profiling between salt-tolerant Pokkali and salt-sensitive Pusa Basmati with or without salt treatment, Goswami et al., found that the expression levels of 65 miRNAs in Pokkali grown under control condition and in Pusa Basmati grown under salt treatment were similar [76]. The salt-induced dysregulations in miRNAs expression profiles showed controlled variations in Pokkali, but larger changes were seen in Pusa Basmati. Further target analysis of salt-deregulated miRNAs by degradome sequencing observed key transcription factors, ion transporters, and signaling molecules that maintain cellular Ca^2+^ balance and inhibit ROS production, thereby enhancing the salt tolerance of rice [76].

## 4. Monitoring Response to Salt Stress by Proteomics

Although most stress-responsive genes are regulated at the transcriptional level, in some cases, changes at the transcriptional level are not always accompanied with the changes in protein abundance due to the translation and post-translational regulation. In addition, protein is the ultimate functional executor of an organism. Therefore, the analysis of proteome is of great significance, and proteomics is developed on this basis. Proteomics refers to the study of protein characteristics at a high-throughput level, including protein expression level, post-translational modification, protein-protein interaction, etc.

The commonly used quantitative proteomics based on mass spectrometry can be divided into two categories: one is stable isotope-labeled quantitative proteomics, such as iTRAQ; The other is unlabeled quantitative proteomics technique, such as label-free. Proteomics is an effective tool for systematically studying the complex mechanisms of salt stress response in rice.

The normal performance of cell functions requires coordination among different cellular compartments and organelles. Salt stress causes cellular damage, resulting in stress on various organelles such as the cell wall, chloroplast, mitochondria, and endoplasmic reticulum. The plant cell wall is composed of cellulose, hemicellulose, pectin, and many glycoproteins, and its integrity is an important factor in determining salt tolerance [1]. Using a proteomic approach, Sengupta and Majumder found that more than 400 protein spots in rice germplasms with different salt tolerance changed significantly under salinity [77]. Among these differentially expressed proteins, SSP8908, a highly upregulated spot in the wild halophytic rice (*Porteresia coarctata*) only under 400 mM NaCl stress, was identified as a cellulose synthase-like protein. SSP8908 may be an important tool to regulate the cellulose synthesis of the cell wall in wild rice, thus further improving the salt tolerance of wild rice. Ubiquitination is an important post-translational modification prevalent in plants, and plays an important role in regulating stress response and other physiological functions. Based on a proteomic comparison of salt-responsive ubiquitin-related proteins in rice roots of cultivar TNG67 and its NaN3-induced pure mutants, SM75 (salt-tolerant) and SA0604 (salt-sensitive), Liu et al., found that ubiquitin modified cellulose synthase A was increased in TNG67 and SM75 under salt stress, whereas it was reduced in SA0604 after salt stress. Moreover, the salt tolerance of those three lines was consistent with the abundance of ubiquitinated cellulose synthase A under salt stress [78], which further supports the vital role of cellulose or cell wall in rice salt tolerance.

Salt stress has multiple harmful effects on chloroplasts, including reduced CO_2_ uptake due to stomatal closure, decreased photosynthetic efficiency, oxidative stress, damaged thylakoid membrane, disturbed osmotic and ionic homeostasis, and impaired protein synthesis and turnover. The reduced photosynthesis efficiency is a major reason for growth inhibition under high salinity. As for rice, the photosystem II (PSII) oxygen evolving complex protein under salt stress was identified through amino acid sequencing after separated by the combination of two-dimensional polyacrylamide gel electrophoresis (2-DE) and CBB-staining [79]. A comparison of 2-DE protein profiles between the control and salt-stressed rice seedlings revealed 55 differentially expressed CBB-stained protein spots [80]. Among these changed spots, the identity of 33 spots were determined by nESI-LC-MS/MS, most of which were involved in the photosynthetic process that is probably responsible for the morphological changes in rice seedlings. Hosseini et al., compared the proteome of two closely related rice genotypes with contrasting responses to salt stress, and found significant down-regulation of proteins associated with photosynthetic electron transport in salt-sensitive IR29 [81], suggesting that photosynthesis was influenced during salt stress. In contrast, the abundance of superoxide dismutase, fibre protein, ferredoxin thioredoxin reductase, and inorganic pyrophosphatase, which may function in salt tolerance, were up-regulated in salt-tolerant FL478. Xu et al., analyzed proteomic changes in rice shoots under salt stress using iTRAQ approach, and found that six significantly changed proteins were enriched in the pathways of photosynthesis, including photosystem I reaction center subunit II precursor-like protein (PsaD), photosystem-1 H subunit GOS5 (PsaH), putative chloroplast chaperonin, and three light-harvesting antenna complex I (LHCI) subunits [82]. These data suggest the important role of photosynthesis in balancing energy supply and salt stress.

The mitochondrion is an energy-producing organelle that is crucial for the survival of plants under multiple environmental stresses. Abiotic stress-induced mitochondrial dysfunction can activate the expression of stress-responsive genes through the mitochondrial retrograde signaling pathway. As for rice, the proteomic profiles from several reports revealed that glycine decarboxylase (GDC) and glycine hydroxymethyltransferase (SHMT) in the photorespiratory pathway were up-regulated under salt stress [80]; mitochondrial prohibiting complex protein 1 and putative mitochondrial processing peptidase related to protein synthesis and assembly were down-regulated in salt-sensitive IR64 under salt stress [83]; fumarate hydratase 1, formate dehydrogenase 1, ATP synthase, and succinyl-CoA ligase involved in the tricarboxylic acid cycle were changed under salt stress [84,85].

Salt-induced production of ROS occurs in the apoplast, chloroplasts, mitochondria, and peroxisomes. An excessive accumulation of ROS under salt stress has adverse effects on plant tissues. To alleviate the oxidative stress caused by excessive ROS accumulation under high salinity, plants depend on the activation of ROS-scavenging machinery, including non-enzymatic antioxidant metabolites and enzymatic agents. The enzymatic agents are composed of catalases (CAT), superoxide dismutase (SOD), peroxidase (POD), ascorbate peroxidase (APX), glutathione peroxidase (GPX), and glutathione reductase (GR). In rice, these enzymes are usually induced by high salinity, which is demonstrated by the proteomic profiles of several reports. For example, Parker et al., found that the SOD content was increased in the rice leaf after salt treatment for 7 days [86]. A comparative proteomic analysis of the salt-tolerant and sensitive rice seedlings after exposure to 300 mM NaCl revealed that APX and Class III POD were increased in salt-tolerant rice after one day of salt stress, while in salt-sensitive rice they were down-regulated or not changed [87]. Proteome profiling by Mishra et al., also confirmed higher expression of SOD, POD, and other proteins in the salt-tolerant lines than those in salt-sensitive lines [88]. Similarly, based on the proteome data, the levels of CAT, POD, APX, GPX, and GR were higher in *sd58* mutant with enhanced salt tolerance than those in its wild type, which was consistent with the lower accumulation of ROS in *sd58* than that in wild type [89].

## 5. Metabolomics Reveals the Metabolic Pathways Involved in Salt Stress

Plants need to produce a large number of metabolites (molecular mass < 1000) during their growth and development to cope with the changing environment and various stresses. The metabolome is made up of these metabolites. Compared with other omics, metabolome is more closely related to the physiological and biochemical state of organisms, and thus plays an important role in modern plant biology research. Metabolomics is a high-throughput metabolome detection technology for simultaneous qualitative and quantitative analysis of a huge arsenal of metabolites that act as substrates and products of various metabolic pathways in cells or organisms during a specific physiological period. The most common metabolomics analysis methods include mass spectrometry (MS) and nuclear magnetic resonance (NMR). Metabolomics has been widely applied to plant biology, especially in rice [90], but there are few studies on rice metabolomics under salt stress.

Siahpoosh et al., investigated the metabolic depletion syndrome in roots of several salt-sensitive rice cultivars under salt stress by physiological and metabolomic approaches, and observed a strong reduction of at least 30 primary metabolites including sucrose, fructose, glucose, fructose-6P, glucose-6-P, organic and amino-acids in rice roots after prolonged exposure to high-salt concentrations. Based on these observations, they hypothesized that sucrose allocation to the root may modify the salt response of rice [91]. Zhao et al., surveyed the metabolic profiles of two rice genotypes with contrasting salt stress tolerance at the seedling stage, and found that their metabolites were tissue-specifically and genotype-dependently regulated under salt stress [92]. Compared with the salt-sensitive cultivar IR64, the salt-tolerant variety FL478 exhibited greater increases in amino acids and sugars and more decreases in organic acids in both leaves and roots. In addition, the maximum change in amino acids and sugars occurred during the later salt-stress stage, while organic acids changed at an early stage. Kusuda et al., compared metabolite profiling of both *MIPS*-overexpressing and wild-type rice, and found that overexpression of the *MIPS* gene enhances salt tolerance by activation of a set of basal metabolisms, such as glycolysis, the tricarboxylic acid cycle, the pentose phosphate pathway, and inositol metabolism [93]. Nam et al., performed metabolite profiling in 38 rice germplasms that varied in biomass accumulation under long-term mild salinity conditions. They observed that the changes of allantoin and glutamine metabolites were positively correlated with the growth potential and salt tolerance of rice [94]. By comparing the abundance of 91 metabolites in two salt-sensitive and two salt-tolerant varieties, Gupta et al., revealed that the increased levels of serotonin and gentisic acid in leaves of tolerant varieties contributed to their salt tolerance [95]. In a comprehensive analysis of the physiological and metabolite changes in rice plants from salt stress, Ma et al., found that γ-amino butyric acid, acetic acid, and sucrose were up-regulated in salt-tolerant lines, while glutamine and putrescine were higher in salt-sensitive lines [96]. Chang et al., compared the metabolites of three rice varieties with different salt tolerance under salt stress, and found that the responses of metabolites to salt stress were time-, tissue- and cultivar-dependent [97]. For example, the contents of shikimate and quinate, involved in the shikimate pathway, were reduced in leaves of all used varieties. The sucrose was significantly up-regulated while malate was significantly down-regulated only in the leaves of salt-tolerant varieties. In addition, some sugars in the leaves of the salt-tolerant cultivars showed earlier increases under salt stress compared to salt-sensitive varieties. Chen et al., determined the metabolome profile of Dongxiang wild rice, and demonstrated the important role of amino acids in rice salt stress [98]. By comparing the differential metabolites of salt-tolerant and salt-sensitive progenies from the backcross inbred lines (BC_1_F_9_) between Dongxiang (donor parent) and cultivated rice R974 (recurrent parent), they further revealed that the asparagine content may be used as one of the positive indexes to evaluate the salt tolerance of rice. A similar result was also found by Ma et al. [99], which exhibited an upregulation of asparagine in a salt-tolerant rice genotype.

**Figure 2:**
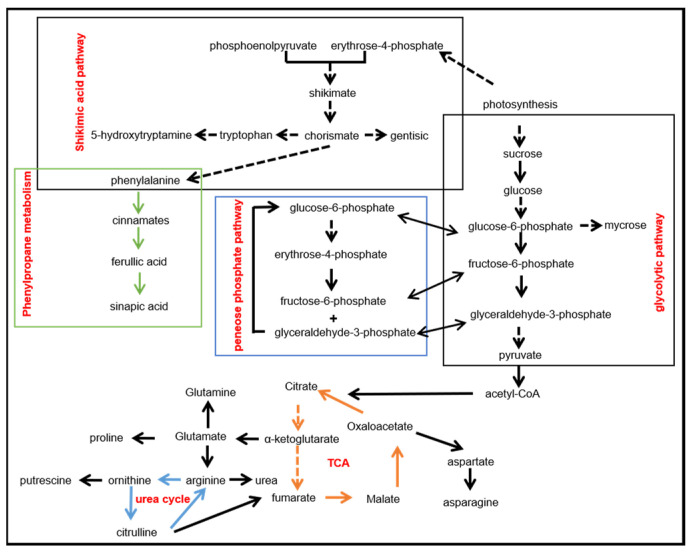
Metabolic pathways involved in salt stress. Blue numbers indicate related references.

In summary, to provide an overview of these metabolites and their metabolic pathways involved in salt stress, we drew a map as shown in Figure 2. It shows that salt stress is directly associated with the shikimic acid pathway, pentose phosphate pathway, glycolysis pathway, tricarboxylic acid cycle, and ornithine cycle, which play a key role in maintaining osmotic balance and affecting plant growth and development.

## 6. Changes in Global DNA Methylation State Induced by Salt Stress

DNA methylation is an epigenetic modification that affects plant development and response to environmental stresses by modulating gene expression, chromosome activation, genomic imprinting, and protection of genomes from invading transposons, retrotransposons, and viruses [100]. DNA methylation in plants occurs in CG, CHH, and CHG (H = A, T, or C) contexts through different pathways. The level of DNA methylation levels is regulated by a complex interplay of DNA methyltransferases, DNA demethylases, and other mechanisms such as the RNA-directed DNA methylation (RdDM) pathway [101]. However, the functional consequences of DNA methylation under salt stress in rice are still largely unknown. Karan et al., studied the effect of salt stress on the degree and pattern of DNA methylation in four rice genotypes with different salt tolerance using the methylation-sensitive amplification polymorphism (MSAP) technique [102]. They revealed that the changes of cytosine methylation and gene expression in rice under salt stress varied with genotypes and tissue types, regardless of the level of salt tolerance of rice genotypes. However, Wang et al., used the same technique to compare DNA methylation changes in two rice genotypes with contrasting salt tolerance and found that genotypic specificity of the detected DNA methylation sites was no more than 10.5% [103]. Ferreira et al., evaluated the global DNA methylation levels in salt-sensitive (IR29) and salt-tolerant (Pokkali) rice varieties under salt stress using the 5-methylcytosine (5mC) antibody and an ELISA based method. They found that the variations of overall DNA methylation levels in response to salt stress were genotype- and tissue-dependent, and the higher DNA methylation adjustment levels were associated with salt tolerance. Specifically, salt stress induced the DNA demethylation of ‘Pokkali’ due to increased expression of DNA demethylases, while ‘IR29’ showed lower plasticity of DNA demethylation due to simultaneous induction of DNA demethylases and methyltransferases [104]. Garg et al., characterized DNA methylation patterns and their effects on transcription in three rice cultivars (IR64, stress-sensitive; Pokkali, salt-tolerant; Nagina 22, drought-tolerant) under normal growth conditions through a combined analysis of whole-genome bisulphite sequencing and RNA sequencing. Overall, there were significant differences in methylation levels among the three rice cultivars, and many differential methylation regions (DMRs) among different cultivars were associated with differential expression of important genes for abiotic stress response [105]. To understand the role of DNA methylation in response to desiccation and salt stresses, they further analyzed the role of DNA methylation under desiccation and salt stresses in these rice cultivars via bisulphite sequencing. The results showed that most of the differentially methylated and differentially expressed genes (DMR-DEGs) were cultivar-specific, suggesting a key role of DNA methylation in a cultivar-specific manner for rice abiotic stress responses [106]. Plant polyploidy can trigger genetic and epigenetic changes that enhance the adaptive potential of plants under extreme environments, but the molecular basis for this is unclear. By comparing diploid and autotetraploid rice under control and salinity conditions, Wang et al., found that salt tolerance of tetraploid rice was enhanced by reducing sodium uptake, which was correlated with epigenetic regulation of jasmonic acid (JA) related genes [107]. In detail, tetraploidy promoted DNA hypomethylation and enhanced genomic loci coexistent with multiple stress-responsive genes including those in jasmonate biosynthesis and signaling pathways, which are usually associated with proximal transposable elements (TEs). Under salt stress, induced expression of salt-responsive genes can enhance hypermethylation and inhibit adjacent TEs. Based on the experimental evidence, they proposed a feedback regulation between polyploidy-induced DNA hypomethylation in fast and robust stress response and stress-induced hypermethylation to repress TEs and TE-associated genes, and speculated that this feedback regulation gives an evolutionary advantage for selection to enhance adaptation in polyploid plants and crops.

## 7. Challenges and Perspectives

Salt tolerance is a multi-genic trait that involves a complex of responses at metabolic, molecular, cellular, physiological, and whole-plant levels. Although numerous salt-responsive QTLs/QTNs and even genes have been isolated and validated from different rice genotypes due to the development and application of omics technology, there are still many limitations. For instance, most of the omics-based studies on salt stress in rice were focused on the mining of QTLs/QTNs, DEGs, differential proteins and metabolites, but relatively few genes have been identified and validated experimentally. One of the main reasons for this phenomenon is that these studies often used only one omics approach, making it difficult to narrow the target underlying salt tolerance. Omics’ approaches are interrelated and dependent on each other, thereby integration of multiple omics approaches is necessary to reach an ultimate step i.e., identification of key stress-responsive genes and introduction of those genes for generation of improved salt-tolerant rice cultivars. With the rapid development of omics technology and the continuous reduction of sequencing cost, multi-omics association analysis, such as BSA-seq and transcriptome [28,29], GWAS and transcriptome [108], transcriptome and proteome [60], transcriptome and metabolome [63,68,109], have been applied to the study of the salt response of rice in recent years, by which the range of candidate targets was significantly reduced. However, there is currently no multi-omics integration analysis with three or more methods for salt stress in rice, which may have better results. In addition to calling for more joint multi-omics analysis on rice salt stress response, the integration of existing omics data is also an alternative plan. The rapid development of ‘omics’ research has led to the generation of more and more data sets. The main trouble for integration of these huge data sets occurred from the partial and different forms of information accessible on bioinformatics data sources. Therefore, algorithmic methods, such as meta-analysis, have been planned to solve this kind of obstacle. Furthermore, many servers have been set up to allow the integration of high-throughput data, and these servers are also able to display the outcomes in a meaningful biological pathway.

Our knowledge on salt stress signaling in rice is fragmentary, cell-specific and tissue-specific approaches are needed to understand the spatial and temporal complexities of the dynamic molecular responses to salt stress. The recent emergence of single-cell omics offers a solution. The combined molecular and chemical approaches may be insufficient to protect plants from a high salinity environment. Plants in nature live together with a variety of rhizosphere soil microbes. Root exudates that are influenced by plant genotypes and environmental stress conditions play a vital role in determining the composition of rhizosphere microbial communities (also known as rhizosphere microbiome) [110]. Some beneficial rhizosphere microbes can improve plant tolerance to abiotic stresses [111,112], but the underlying molecular mechanisms remain unclear. Exploring and deciphering the protective effects provided by beneficial microbes using microbiome provides a new and promising strategy for improving the salt tolerance of rice.

The accurate and standardized identification of plant phenotypes, especially physiological phenotypes, is important for omics analysis. Compared to the rapid advance of genotyping techniques, the development of phenotyping techniques has undoubtedly lagged, as artificial-based observations still predominate, which becomes a bottleneck for the further development of omics in the future. In addition, this manual method is time-consuming, labor-intensive, and destructive. The difficulties in precisely identifying crop phenotypes are mainly reflected in two aspects. First, plant phenotypic changes are spatially and temporally dependent that vary in different tissues and developmental periods. Second, phenotypes are easily influenced by external environmental conditions, such as light, temperature, humidity, and soil nutrient content. Therefore, accurate phenotyping requires the establishment of a rigorous process that involves defining developmental periods and observing tissues, as well as controlling environmental conditions such as photoperiod, moisture, and temperature. It is almost impossible to develop an accurate phenotypic criterion depending on manual observation, resulting in the fact that most phenotypic data from different studies are hardly comparable. Fortunately, advances in sensor, imaging, and automation technologies have facilitated the creation of automated, intelligent, and high-throughput plant phenotyping platforms, which allow standardized and large-scale identification of plant phenotypes, even those that are susceptible to environmental change. All of these obtained phenotypes from the plant phenotyping platform are called the phenome. Current progress in phenomics has allowed for a higher resolution of nondestructive assessments of plant responses to salinity throughout the growing season, which will greatly improve the efficiency of the genetic anatomy of salt tolerance traits in rice.

Cultivation of salt-tolerant varieties is the ultimate goal of rice salt tolerance. So far, plant breeding has achieved a leap from artificial selection breeding and hybrid breeding to molecular breeding. Through years of efforts, some salt-tolerant varieties with better comprehensive properties were also bred. For example, two super rice lines with high-yield and salt-tolerant were bred under the genetic background of Pusa44 and Sarjoo52 by integrating Saltol with microsatellite makers [113]. However, in order to meet the food needs of the growing population, people call for new innovations in plant breeding, that is, the breeding 4.0 stage of combining excellent alleles with precise design [114]. For example, the most advanced high-throughput phenotypic technology was used in the EU Horizon 2020 research project NEURICE to evaluate the salt tolerance of rice and assist the breeding of salt-tolerant varieties [11]. Moreover, with the development of mathematics and computer science, we are beginning to enter the age of artificial intelligence (AI), which could lead to another biological revolution [114]. AI technology can be used to mine and integrate massive amounts of omics data more efficiently, such as omics data extraction, genome selection (GS), protein structure prediction, and panomics data integration [115,116,117,118]. Advances in AI, omics, and molecular biology including gene editing allow us to predict salt resistance in rice, and these key genes to precision breeding to improve salt tolerance of rice and support its growth in high salinity soil.

## Figures and Tables

**Table 1:** Summary of salt stress related loci identified by GWAS.

Stage	Population Size	Maker Number	Candidate Site	Reference
Germination stage	478	6,361,920	11	[31]
	371	162,529	56	[32]
	295	1,650,000	12	[33]
	208	395,553	20	[34]
Seedling stage	553	700,000	11	[35]
	295	1,650,000	25	[36]
	235	30,000	73	[37]
	176	68,786	26	[38]
	306	700,000	1900	[39]
	181	32,315	54	[40]
	162	3,000,000	9	[41]
	664	68,376	21	[42]
	204	2,000,000	160	[43]
	155	37,867	151	[44]
	96	50,000	23	[45]
	82	3,340,000	6	[46]
	179	21,623	26	[51]
	92	2,320,000	20	[52]
Reproductive stage	220	6000	20	[47]
	104	112,565	200	[48]
	708	3,455,952	2038	[49]
	180	127	28	[50]

## Data Availability

All data are available upon reasonable request.

## Abbreviations

ROS	Reactive oxygen species
QTL	Quantitative trait locus
BSA	Bulked segregant analysis
RIL	Recombinant inbred lines
ORF	Open reading frame
GWAS	Genome-wide association study
QTN	Quantitative traits nucleotides
LD	Linkage disequilibrium
MATE	Multidrug and toxic compound extrusion
CSSL	Chromosomal segment substitution line
URMC	USDA rice mini-core collection
MATs	Marker-trait associations
AS	Alternative splicing
GSS	Genotype-specific splicing
H_2_O_2_	Hydrogen peroxide
ABA	Abscisic acid
BR	Brassinolide
DEGs	Differentially expressed genes
siRNAs	Short interfering RNAs
miRNAs	MicroRNAs
nat-siRNAs	Nature antisense transcripts-derived siRNAs
ta-siRNAs	Trans-acting siRNAs
lsiRNAs	Long siRNAs
hc-siRNAs	Heterochromatic siRNAs
pre-miRNA	miRNA precursor
RISC	RNA-induced silencing complex
NGS	Next-generation sequencing
LHCI	Light-harvesting antenna complex I
GDC	Glycine decarboxylase
SHMT	Glycine hydroxymethyltransferase
CAT	Catalases
SOD	Superoxide dismutase
POD	Peroxidase
APX	Ascorbate peroxidase
GPX	Glutathione peroxidase
GR	Glutathione reductase
MS	Mass spectrometry
NMR	Nuclear magnetic resonance
RdDM	RNA-directed DNA methylation
MSAP	Methylation-sensitive amplification polymorphism
5mC	5-methylcytosine
DMRs	Differential methylation regions
JA	Jasmonic acid
TEs	Transposable elements
AI	Artificial intelligence
GS	Genome selection

## References

[1] Liu C.T., Mao B.G., Yuan D.Y., Chu C.C., Duan M.J. (2021). Salt tolerance in rice: Physiological responses and molecular mechanisms. Crop J..

[2] Park H.J., Kim W., Yun D. (2016). A new insight of salt stress signaling in plant. Mol. Cells.

[3] Van Zelm E., Zhang Y.X., Testerink C. (2020). Salt tolerance mechanisms of plants. Annu. Rev. Plant Biol..

[4] Zhao C.Z., Zhang H., Song C.P., Zhu J.K., Shabala S. (2020). Mechanisms of plant responses and adaptation to soil salinity. Innovation.

[5] Singh A. (2021). Soil salinization management for sustainable development: A review. J. Environ. Manag..

[6] Gong Z.Z., Xiong L.M., Shi H.Z., Yang S.H., Herrera-Estrella L.R., Xu G.H., Chao D.Y., Li J.R., Wang P.Y., Qin F. (2020). Plant abiotic stress response and nutrient use efficiency. Sci. China Life Sci..

[7] Munns R., Tester M. (2008). Mechanisms of salinity tolerance. Annu. Rev. Plant Biol..

[8] Jha U.C., Bohra A., Jha R., Parida S.K. (2019). Salinity stress response and ‘omics’ approaches for improving salinity stress tolerance in major grain legumes. Plant Cell Rep..

[9] Zeng L., Shannon M., Grieve C. (2002). Evaluation of salt tolerance in rice genotypes by multiple agronomic parameters. Euphytica.

[10] Ponce K.S., Guo L.B., Leng Y.J., Meng L.J., Ye G.Y. (2021). Advances in sensing, response and regulation mechanism of salt tolerance in rice. Int. J. Mol. Sci..

[11] Ganie S.A., Wani S.H., Henry R., Hensel G. (2021). Improving rice salt tolerance by precision breeding in a new era. Curr. Opin. Plant Biol..

[12] Fan X.R., Jiang H.Z., Meng L.J., Chen J.G. (2021). Gene mapping, cloning and association analysis for salt tolerance in rice. Int. J. Mol. Sci..

[13] Qin H., Li Y.X., Huang R. (2020). Advances and challenges in the breeding of salt-tolerant rice. Int. J. Mol. Sci..

[14] Ren Z.H., Gao J.P., Li L.G., Cai X.L., Huang W., Chao D.Y., Zhu M.Z., Wang Z.Y., Luan S., Lin H.X. (2005). A rice quantitative trait locus for salt tolerance encodes a sodium transporter. Nat. Genet..

[15] Fukuda A., Nakamura A., Tagiri A., Tanaka H., Miyao A., Hirochika H., Tanaka Y. (2004). Function, intracellular localization and the importance in salt tolerance of a vacuolar Na^+^/H^+^ antiporter from rice. Plant Cell Physiol..

[16] Martínez-Atienza J., Jiang X., Garciadeblas B., Mendoza I., Zhu J.K., Pardo J.M., Quintero F.J. (2007). Conservation of the salt overly sensitive pathway in rice. Plant Physiol..

[17] El Mahi H., Pérez-Hormaeche J., De Luca A., Villalta I., Espartero J., Gámez-Arjona F., Fernández J.L., Bundó M., Mendoza I., Mieulet D. (2019). A critical role of sodium flux via the plasma membrane Na^+^/H^+^ exchanger SOS1 in the salt tolerance of rice. Plant Physiol..

[18] Li J., Long Y., Qi G.N., Xu Z.J., Wu W.H., Wang Y. (2014). The Os-AKT1 channel is critical for K^+^ uptake in rice roots and is modulated by the rice CBL1-CIPK23 complex. Plant Cell.

[19] Tian Q.X., Shen L.K., Luan J.X., Zhou Z.Z., Guo D.S., Shen Y., Jing W., Zhang B.L., Zhang Q., Zhang W.H. (2021). Rice shaker potassium channel OsAKT2 positively regulates salt tolerance and grain yield by mediating K^+^ redistribution. Plant Cell Env..

[20] Huang Y.N., Yang S.Y., Li J.L., Wang S.F., Wang J.J., Hao D.L., Su Y.H. (2021). The rectification control and physiological relevance of potassium channel OsAKT2. Plant Physiol..

[21] Huang L.K., Tang W.Q., Wu W.R. (2022). Optimization of BSA-seq experiment for QTL mapping. G3-Genes Genom. Genet..

[22] Zegeye W.A., Zhang Y.X., Cao L.Y., Cheng S.H. (2018). Whole Genome Resequencing from bulked populations as a rapid QTL and gene identification method in rice. Int. J. Mol. Sci..

[23] Zou C., Wang P.X., Xu Y.B. (2016). Bulked sample analysis in genetics, genomics and crop improvement. Plant Biotechnol. J..

[24] Abe A., Kosugi S., Yoshida K., Natsume S., Takagi H., Kanzaki H., Matsumura H., Yoshida K., Mitsuoka C., Tamiru M. (2012). Genome sequencing reveals agronomically important loci in rice using MutMap. Nat. Biotechnol..

[25] Takagi H., Uemura A., Yaegashi H., Tamiru M., Abe A., Mitsuoka C., Utsushi H., Natsume S., Kanzaki H., Matsumura H. (2013). MutMap-Gap: Whole-genome resequencing of mutant F2 progeny bulk combined with *de novo* assembly of gap regions identifies the rice blast resistance gene *Pii*. New Phytol..

[26] Takagi H., Tamiru M., Abe A., Yoshida K., Uemura A., Yaegashi H., Obara T., Oikawa K., Utsushi H., Kanzaki E. (2015). MutMap accelerates breeding of a salt-tolerant rice cultivar. Nat. Biotechnol..

[27] Tiwari S., Sl K., Kumar V., Singh B., Rao A., Mithra SV A., Rai V., Singh A.K., Singh N.K. (2016). Mapping QTLs for salt tolerance in rice (*Oryza sativa* L.) by bulked segregant analysis of recombinant inbred lines using 50K SNP chip. PLoS ONE.

[28] Sun B.R., Fu C.Y., Fan Z.L., Chen Y., Chen W.F., Zhang J., Jiang L.Q., Lv S., Pan D.J., Li C. (2019). Genomic and transcriptomic analysis reveal molecular basis of salinity tolerance in a novel strong salt-tolerant rice landrace Changmaogu. Rice.

[29] Lei L., Zheng H.L., Bi Y.L., Yang L.M., Liu H.L., Wang J.G., Sun J., Zhao H.W., Li X.W., Li J.M. (2020). Identification of a major qtl and candidate gene analysis of salt tolerance at the bud burst stage in rice (*Oryza sativa* L.) using QTL-Seq and RNA-Seq. Rice.

[30] Wu F.L., Yang J., Yu D.Q., Xu P. (2020). Identification and validation a major QTL from “Sea Rice 86” seedlings conferred salt tolerance. Agronomy.

[31] Shi Y.Y., Gao L.L., Wu Z.C., Zhang X.J., Wang M.M., Zhang C.S., Zhang F., Zhou Y.L., Li Z.K. (2017). Genome-wide association study of salt tolerance at the seed germination stage in rice. BMC Plant Biol..

[32] Cui Y.R., Zhang F., Zhou Y.L. (2018). The application of multi-locus GWAS for the detection of salt-tolerance loci in rice. Front. Plant Sci..

[33] Yu J., Zhao W.G., Tong W., He Q., Yoon M.Y., Li F.P., Choi B., Heo E.B., Kim K.W., Park Y.J. (2018). A genome-wide association study reveals candidate genes related to salt tolerance in rice (*Oryza sativa*) at the germination stage. Int. J. Mol. Sci..

[34] Naveed S.A., Zhang F., Zhang J., Zheng T.Q., Meng L.J., Pang Y.L., Xu J.L., Li Z.K. (2018). Identification of QTN and candidate genes for salinity tolerance at the germination and seedling stages in rice by genome-wide association analyses. Sci. Rep..

[35] Meyer R.S., Choi J.Y., Sanches M., Plessis A., Flowers J.M., Amas J., Dorph K., Barretto A., Gross B., Fuller D.Q. (2016). Domestication history and geographical adaptation inferred from a SNP map of African rice. Nat. Genet..

[36] Yu J., Zao W.G., He Q., Kim T.S., Park Y.J. (2017). Genome-wide association study and gene set analysis for understanding candidate genes involved in salt tolerance at the rice seedling stage. Mol. Genet. Genom..

[37] Frouin J., Languillaume A., Mas J., Mieulet D., Boisnard A., Labeyrie A., Bettembourg M., Bureau C., Lorenzini E., Portefaix M. (2018). Tolerance to mild salinity stress in japonica rice: A genome-wide association mapping study highlights calcium signaling and metabolism genes. PLoS ONE.

[38] Batayeva D., Labaco B., Ye C.R., Li X.L., Usenbekov B., Rysbekova A., Dyuskalieva G., Vergara G., Reinke R., Leung H. (2018). Genome-wide association study of seedling stage salinity tolerance in *Temperate japonica* rice germplasm. BMC Genet..

[39] Patishtan J., Hartley T.N., Fonseca de Carvalho R., Maathuis F.J. (2018). Genome-wide association studies to identify rice salt-tolerance markers. Plant Cell Environ..

[40] An H.Z., Liu K., Wang B.X., Tian Y.L., Ge Y.W., Zhang Y.Y., Tang W.J., Chen G.M., Yu J., Wu W. (2020). Genome-wide association study identifies QTLs conferring salt tolerance in rice. Plant Breed..

[41] Rohila J.S., Edwards J.D., Tran G.D., Jackson A.K., McClung A.M. (2019). Identification of superior alleles for seedling stage salt tolerance in the USDA rice mini-core collection. Plants.

[42] Yuan J., Wang X.Q., Zhao Y., Khan N.U., Zhao Z.Q., Zhang Y.L., Wen X.R., Tang F., Wang F.B., Li Z.C. (2020). Genetic basis and identification of candidate genes for salt tolerance in rice by GWAS. Sci. Rep..

[43] Chen C.J., Norton G.J., Price A.H. (2020). Genome-wide association mapping for salt tolerance of rice seedlings grown in hydroponic and soil systems using the bengal and assam aus panel. Front. Plant Sci..

[44] Nayyeripasand L., Garoosi G.A., Ahmadikhah A. (2021). Genome-wide association study (GWAS) to identify salt-tolerance QTLs carrying novel candidate genes in rice during early vegetative stage. Rice.

[45] Yadav A.K., Kumar A., Grover N., Ellur R.K., Bollinedi H., Krishnan S.G., Bhowmick P.K., Vinod K.K., Nagarajan M., Singh A.K. (2021). Genome-wide association study reveals marker–trait associations for early vegetative stage salinity tolerance in rice. Plants.

[46] Yu H.H., Du Q., Campbell M., Yu B., Walia H., Zhang C. (2021). Genome-wide discovery of natural variation in pre-mRNA splicing and prioritising causal alternative splicing to salt stress response in rice. New Phytol..

[47] Kumar V., Singh A., Mithra S.A., Krishnamurthy S., Parida S.K., Jain S., Tiwari K.K., Kumar P., Rao A.R., Sharma S.K. (2015). Genome-wide association mapping of salinity tolerance in rice (*Oryza sativa*). DNA Res..

[48] Lekklar C., Pongpanich M., Suriya-Arunroj D., Chinpongpanich A., Tsai H., Comai L., Chadchawan S., Buaboocha T. (2019). Genome-wide association study for salinity tolerance at the flowering stage in a panel of rice accessions from Thailand. BMC Genom..

[49] Liu C., Chen K., Zhao X.Q., Wang X.Q., Shen C.C., Zhu Y.J., Dai M.L., Qiu X.J., Yang R.W., Xing D.Y. (2019). Identification of genes for salt tolerance and yield-related traits in rice plants grown hydroponically and under saline field conditions by genome-wide association study. Rice.

[50] Warraich A.S., Krishnamurthy S., Sooch B.S., Vinaykumar N., Dushyanthkumar B., Bose J., Sharma P.C. (2020). Rice GWAS reveals key genomic regions essential for salinity tolerance at reproductive stage. Acta Physiol. Plant..

[51] Le T.D., Gathignol F., Vu H.T., Nguyen K.L., Tran L.H., Vu H.T.T., Dinh T.X., Lazennec F., Pham X.H., Véry A.A. (2021). Genome-wide association mapping of salinity tolerance at the seedling stage in a panel of Vietnamese landraces reveals new valuable QTLs for salinity stress tolerance breeding in rice. Plants.

[52] Al-Tamimi N., Brien C., Oakey H., Berger B., Saade S., Ho Y.S., Schmöckel S.M., Tester M., Negrão S. (2016). Salinity tolerance loci revealed in rice using high-throughput non-invasive phenotyping. Nat. Commun..

[53] Zhang H.M., Zhu J.H., Gong Z.Z., Zhu J.K. (2021). Abiotic stress responses in plants. Nat. Rev. Genet..

[54] Hossain M.R., Bassel G.W., Pritchard J., Sharma G.P., Ford-Lloyd B.V. (2016). Trait specific expression profiling of salt stress responsive genes in diverse rice genotypes as determined by modified significance analysis of microarrays. Front. Plant Sci..

[55] Mirdar Mansuri R., Shobbar Z.S., Babaeian Jelodar N., Ghaffari M.R., Nematzadeh G.A., Asari S. (2019). Dissecting molecular mechanisms underlying salt tolerance in rice: A comparative transcriptional profiling of the contrasting genotypes. Rice.

[56] Fu S., Fu L.B., Zhang X., Huang J.J., Yang G.Z., Wang Z.G., Liu Y.G., Zhang G.Q., Wu D.Z., Xia J.X. (2019). OsC2DP, a novel C2 domain-containing protein is required for salt tolerance in rice. Plant Cell Physiol..

[57] Cotsaftis O., Plett D., Johnson A.A., Walia H., Wilson C., Ismail A.M., Close T.J., Tester M., Baumann U. (2011). Root-specific transcript profiling of contrasting rice genotypes in response to salinity stress. Mol. Plant.

[58] Wei H., Wang X.L., He Y.Q., Xu H., Wang L. (2021). Clock component OsPRR73 positively regulates rice salt tolerance by modulating *OsHKT2;1*-mediated sodium homeostasis. EMBO J..

[59] Jan A., Maruyama K., Todaka D., Kidokoro S., Abo M., Yoshimura E., Shinozaki K., Nakashima K., Yamaguchi-Shinozaki K. (2013). OsTZF1, a CCCH-tandem zinc finger protein, confers delayed senescence and stress tolerance in rice by regulating stress-related genes. Plant Physiol..

[60] Li Y.F., Zheng Y., Vemireddy L.R., Panda S.K., Jose S., Ranjan A., Panda P., Govindan G., Cui J.X., Wei K.N. (2018). Comparative transcriptome and translatome analysis in contrasting rice genotypes reveals differential mRNA translation in salt-tolerant Pokkali under salt stress. BMC Genom..

[61] Liang C.Z., Zheng G.Y., Li W.Z., Wang Y.Q., Hu B., Wang H.R., Wu H.K., Qian Y.W., Zhu X.G., Tan D.X. (2015). Melatonin delays leaf senescence and enhances salt stress tolerance in rice. J. Pineal Res..

[62] Rabbani M.A., Maruyama K., Abe H., Khan M.A., Katsura K., Ito Y., Yoshiwara K., Seki M., Shinozaki K., Yamaguchi-Shinozaki K. (2003). Monitoring expression profiles of rice genes under cold, drought, and high-salinity stresses and abscisic acid application using cDNA microarray and RNA gel-blot analyses. Plant Physiol..

[63] Wang W.S., Zhao X.Q., Li M., Huang L.Y., Xu J.L., Zhang F., Cui Y.R., Fu B.Y., Li Z.K. (2016). Complex molecular mechanisms underlying seedling salt tolerance in rice revealed by comparative transcriptome and metabolomic profiling. J. Exp. B.

[64] Dong N., Yin W.C., Liu D.P., Zhang X.X., Yu Z.K., Huang W., Liu J.H., Yang Y.Z., Meng W.J., Niu M. (2020). Regulation of brassinosteroid signaling and salt resistance by SERK2 and potential utilization for crop improvement in rice. Front. Plant Sci..

[65] Formentin E., Barizza E., Stevanato P., Falda M., Massa F., Tarkowskà D., Novák O., Lo Schiavo F. (2018). Fast regulation of hormone metabolism contributes to salt tolerance in rice (*Oryza sativa* spp. Japonica, L.) by inducing specific morpho-physiological responses. Plants.

[66] Zhou Y., Yang P., Cui F.L., Zhang F.T., Luo X.D., Xie J.K. (2016). Transcriptome analysis of salt stress responsiveness in the seedlings of Dongxiang wild rice (*Oryza rufipogon* Griff.). PLoS ONE.

[67] Cartagena J.A., Yao Y., Mitsuya S., Tsuge T. (2021). Comparative transcriptome analysis of root types in salt tolerant and sensitive rice varieties in response to salinity stress. Physiol. Plant..

[68] Xie Z.Y., Wang J., Wang W.S., Wang Y.R., Xu J.L., Li Z.K., Zhao X.Q., Fu B.Y. (2021). Integrated analysis of the transcriptome and metabolome revealed the molecular mechanisms underlying the enhanced salt tolerance of rice due to the application of exogenous melatonin. Front. Plant Sci..

[69] Udomchalothorn T., Plaimas K., Sripinyowanich S., Boonchai C., Kojonna T., Chutimanukul P., Comai L., Buaboocha T., Chadchawan S. (2017). *OsNucleolin1-L* expression in Arabidopsis enhances photosynthesis via transcriptome modification under salt stress conditions. Plant Cell Physiol..

[70] Boonchai C., Udomchalothorn T., Sripinyowanich S., Comai L., Buaboocha T., Chadchawan S. (2018). Rice overexpressing *OsNUC1-S* reveals differential gene expression leading to yield loss reduction after salt stress at the booting stage. Int. J. Mol. Sci..

[71] Khrueasan N., Chutimanukul P., Plaimas K., Buaboocha T., Siangliw M., Toojinda T., Comai L., Chadchawan S. (2019). Comparison between the transcriptomes of ‘KDML105′ rice and a salt-tolerant chromosome segment substitution line. Genes.

[72] Sunkar R., Zhou X.F., Zheng Y., Zhang W.X., Zhu J.K. (2008). Identification of novel and candidate miRNAs in rice by high throughput sequencing. BMC Plant Biol..

[73] Barrera-Figueroa B.E., Gao L., Wu Z.G., Zhou X.F., Zhu J.H., Jin H.L., Liu R.Y., Zhu J.K. (2012). High throughput sequencing reveals novel and abiotic stress-regulated microRNAs in the inflorescences of rice. BMC Plant Biol..

[74] Tripathi A., Chacon O., Lata Singla-Pareek S., Sopory S.K., Sanan-Mishra N. (2018). Mapping the microRNA expression profiles in glyoxalase overexpressing salinity tolerant rice. Curr. Genom..

[75] Parmar S., Gharat S.A., Tagirasa R., Chandra T., Behera L., Dash S.K., Shaw B.P. (2020). Identification and expression analysis of miRNAs and elucidation of their role in salt tolerance in rice varieties susceptible and tolerant to salinity. PLoS ONE.

[76] Goswami K., Mittal D., Gautam B., Sopory S.K., Sanan-Mishra N. (2020). Mapping the salt stress-induced changes in the root miRNome in Pokkali rice. Biomolecules.

[77] Sengupta S., Majumder A.L. (2009). Insight into the salt tolerance factors of a wild halophytic rice, Porteresia coarctata: A physiological and proteomic approach. Planta.

[78] Liu C.W., Hsu Y.K., Cheng Y.H., Yen H.C., Wu Y.P., Wang C.S., Lai C.C. (2012). Proteomic analysis of salt-responsive ubiquitin-related proteins in rice roots. Rapid Commun. Mass Spectrom..

[79] Abbasi F.M., Komatsu S. (2004). A proteomic approach to analyze salt-responsive proteins in rice leaf sheath. Proteomics.

[80] Kim D.W., Rakwal R., Agrawal G.K., Jung Y.H., Shibato J., Jwa N.S., Iwahashi Y., Iwahashi H., Kim D.H., Shim I.S. (2005). A hydroponic rice seedling culture model system for investigating proteome of salt stress in rice leaf. Electrophoresis.

[81] Hosseini S.A., Gharechahi J., Heidari M., Koobaz P., Abdollahi S., Mirzaei M., Nakhoda B., Salekdeh G.H. (2015). Comparative proteomic and physiological characterisation of two closely related rice genotypes with contrasting responses to salt stress. Funct. Plant Biol..

[82] Xu J.W., Lan H.X., Fang H.M., Huang X., Zhang H.S., Huang J. (2015). Quantitative proteomic analysis of the rice (*Oryza sativa* L.) salt response. PLoS ONE.

[83] Sarhadi E., Bazargani M.M., Sajise A.G., Abdolahi S., Vispo N.A., Arceta M., Nejad G.M., Singh R.K., Salekdeh G.H. (2012). Proteomic analysis of rice anthers under salt stress. Plant Physiol. Bioch..

[84] Liu C.W., Chang T.S., Hsu Y.K., Wang A.Z., Yen H.C., Wu Y.P., Wang C.S., Lai C.C. (2014). Comparative proteomic analysis of early salt stress responsive proteins in roots and leaves of rice. Proteomics.

[85] Lakra N., Kaur C., Singla-Pareek S.L., Pareek A. (2019). Mapping the ‘early salinity response’ triggered proteome adaptation in contrasting rice genotypes using iTRAQ approach. Rice.

[86] Parker R., Flowers T.J., Moore A.L., Harpham N.V. (2006). An accurate and reproducible method for proteome profiling of the effects of salt stress in the rice leaf lamina. J. Exp. Bot..

[87] Damaris R.N., Li M., Liu Y., Chen X., Murage H., Yang P.A. (2016). Proteomic analysis of salt stress response in seedlings of two African rice cultivars. BBA-Proteins Proteom..

[88] Mishra P., Mishra V., Takabe T., Rai V., Singh N.K. (2016). Elucidation of salt-tolerance metabolic pathways in contrasting rice genotypes and their segregating progenies. Plant Cell Rep..

[89] Peng P., Gao Y.D., Li Z., Yu Y.W., Qin H., Guo Y., Huang R.F., Wang J. (2019). Proteomic analysis of a rice mutant *sd58* possessing a novel *d1* allele of heterotrimeric G protein alpha subunit (RGA1) in salt stress with a focus on ROS scavenging. Int. J. Mol. Sci..

[90] Yang C., Shen S., Zhou S., Li Y., Mao Y., Zhou J., Shi Y., An L., Zhou Q., Peng W. (2022). Rice metabolic regulatory network spanning the entire life cycle. Mol. Plant.

[91] Siahpoosh M.R., Sanchez D.H., Schlereth A., Scofield G.N., Furbank R.T., van Dongen J.T., Kopka J. (2012). Modification of *OsSUT1* gene expression modulates the salt response of rice *Oryza sativa* cv. Taipei 309. Plant Sci..

[92] Zhao X.Q., Wang W.S., Zhang F., Deng J.L., Li Z.K., Fu B.Y. (2014). Comparative metabolite profiling of two rice genotypes with contrasting salt stress tolerance at the seedling stage. PLoS ONE.

[93] Kusuda H., Koga W., Kusano M., Oikawa A., Saito K., Hirai M.Y., Yoshida K.T. (2015). Ectopic expression of *myo*-inositol 3-phosphate synthase induces a wide range of metabolic changes and confers salt tolerance in rice. Plant Sci..

[94] Nam M.H., Bang E., Kwon T.Y., Kim Y., Kim E.H., Cho K., Park W.J., Kim B.G., Yoon I.S. (2015). Metabolite profiling of diverse rice germplasm and identification of conserved metabolic markers of rice roots in response to long-term mild salinity stress. Int. J. Mol. Sci..

[95] Gupta P., De B. (2017). Metabolomics analysis of rice responses to salinity stress revealed elevation of serotonin, and gentisic acid levels in leaves of tolerant varieties. Plant Signal. Behav..

[96] Ma N.L., Che Lah W.A., Abd Kadir N., Mustaqim M., Rahmat Z., Ahmad A., Lam S.D., Ismail M.R. (2018). Susceptibility and tolerance of rice crop to salt threat: Physiological and metabolic inspections. PLoS ONE.

[97] Chang J., Cheong B.E., Natera S., Roessner U. (2019). Morphological and metabolic responses to salt stress of rice (*Oryza sativa* L.) cultivars which differ in salinity tolerance. Plant Physiol. Bioch..

[98] Chen Y.L., Huang W.X., Zhang F.T., Luo X.D., Hu B.L., Xie J.K. (2021). Metabolomic profiling of dongxiang wild rice under salinity demonstrates the significant role of amino acids in rice salt stress. Front. Plant Sci..

[99] Ma N.L., Lam S.D., Lah W.A.C., Ahmad A., Rinklebe J., Sonne C., Peng W. (2021). Integration of environmental metabolomics and physiological approach for evaluation of saline pollution to rice plant. Environ. Pollut..

[100] Sharma R., Mohan Singh R., Malik G., Deveshwar P., Tyagi A.K., Kapoor S., Kapoor M. (2009). Rice cytosine DNA methyltransferases–gene expression profiling during reproductive development and abiotic stress. FEBS J..

[101] Laird P.W. (2010). Principles and challenges of genome-wide DNA methylation analysis. Nat. Rev. Genet..

[102] Karan R., DeLeon T., Biradar H., Subudhi P.K. (2012). Salt stress induced variation in DNA methylation pattern and its influence on gene expression in contrasting rice genotypes. PLoS ONE.

[103] Wang W.S., Huang F., Qin Q., Zhao X.Q., Li Z.K., Fu B.Y. (2015). Comparative analysis of DNA methylation changes in two rice genotypes under salt stress and subsequent recovery. Biochem. Biophys. Res. Commun..

[104] Ferreira L.J., Azevedo V., Maroco J., Oliveira M.M., Santos A.P. (2015). Salt tolerant and sensitive rice varieties display differential methylome flexibility under salt stress. PLoS ONE.

[105] Garg R., Narayana Chevala V., Shankar R., Jain M. (2015). Divergent DNA methylation patterns associated with gene expression in rice cultivars with contrasting drought and salinity stress response. Sci. Rep..

[106] Rajkumar M.S., Shankar R., Garg R., Jain M. (2020). Bisulphite sequencing reveals dynamic DNA methylation under desiccation and salinity stresses in rice cultivars. Genomics.

[107] Wang L.F., Cao S., Wang P.T., Lu K.N., Song Q.X., Zhao F.J., Chen Z.J. (2021). DNA hypomethylation in tetraploid rice potentiates stress-responsive gene expression for salt tolerance. Proc. Natl. Acad. Sci. USA.

[108] Kong W.L., Zhang C.H., Zhang S.C., Qiang Y.L., Zhang Y., Zhong H., Li Y.S. (2021). Uncovering the novel QTLs and candidate genes of salt tolerance in rice with linkage mapping, RTM-GWAS, and RNA-seq. Rice.

[109] Wang Y.X., Huang L.Y., Du F.P., Wang J., Zhao X.Q., Li Z.K., Wang W.S., Xu J.L., Fu B.Y. (2021). Comparative transcriptome and metabolome profiling reveal molecular mechanisms underlying *OsDRAP1*-mediated salt tolerance in rice. Sci. Rep..

[110] Reinhold-Hurek B., Bünger W., Burbano C.S., Sabale M., Hurek T. (2015). Roots shaping their microbiome: Global hotspots for microbial activity. Annu. Rev. phytopathol..

[111] Liu X.M., Zhang H.M. (2015). The effects of bacterial volatile emissions on plant abiotic stress tolerance. Front. Plant Sci..

[112] de Faria M.R., Costa L.S.A.S., Chiaramonte J.B., Bettiol W., Mendes R. (2021). The rhizosphere microbiome: Functions, dynamics, and role in plant protection. Trop. Plant Pathol..

[113] Krishnamurthy S.L., Pundir P., Warraich A.S., Rathor S., Lokeshkumar B.M., Singh N.K., Sharma P.C. (2020). Introgressed Saltol QTL lines improves the salinity tolerance in rice at seedling stage. Front. Plant Sci..

[114] Shen Y.T., Zhou G.A., Liang C.Z., Tian Z.X. (2022). Omics-based interdisciplinarity is accelerating plant breeding. Curr. Opin. Plant Biol..

[115] Baek M., DiMaio F., Anishchenko I., Dauparas J., Ovchinnikov S., Lee G.R., Wang J., Cong Q., Kinch L.N., Schaeffer R.D. (2021). Accurate prediction of protein structures and interactions using a three-track neural network. Science.

[116] Jumper J., Evans R., Pritzel A., Green T., Figurnov M., Ronneberger O., Tunyasuvunakool K., Bates R., Žídek A., Potapenko A. (2021). Highly accurate protein structure prediction with AlphaFold. Nature.

[117] Montesinos-López O.A., Montesinos-López A., Pérez-Rodríguez P., Barrón-López J.A., Martini J.W., Fajardo-Flores S.B., Gaytan-Lugo L.S., Santana-Mancilla P.C., Crossa J. (2021). A review of deep learning applications for genomic selection. BMC Genom..

[118] Reel P.S., Reel S., Pearson E., Trucco E., Jefferson E. (2021). Using machine learning approaches for multi-omics data analysis: A review. Biotechnol. Adv..

